# Nomenclature- and Database-Compatible Names for the Two Ebola Virus Variants that Emerged in Guinea and the Democratic Republic of the Congo in 2014

**DOI:** 10.3390/v6114760

**Published:** 2014-11-24

**Authors:** Jens H. Kuhn, Kristian G. Andersen, Sylvain Baize, Yīmíng Bào, Sina Bavari, Nicolas Berthet, Olga Blinkova, J. Rodney Brister, Anna N. Clawson, Joseph Fair, Martin Gabriel, Robert F. Garry, Stephen K. Gire, Augustine Goba, Jean-Paul Gonzalez, Stephan Günther, Christian T. Happi, Peter B. Jahrling, Jimmy Kapetshi, Gary Kobinger, Jeffrey R. Kugelman, Eric M. Leroy, Gael Darren Maganga, Placide K. Mbala, Lina M. Moses, Jean-Jacques Muyembe-Tamfum, Magassouba N’Faly, Stuart T. Nichol, Sunday A. Omilabu, Gustavo Palacios, Daniel J. Park, Janusz T. Paweska, Sheli R. Radoshitzky, Cynthia A. Rossi, Pardis C. Sabeti, John S. Schieffelin, Randal J. Schoepp, Rachel Sealfon, Robert Swanepoel, Jonathan S. Towner, Jiro Wada, Nadia Wauquier, Nathan L. Yozwiak, Pierre Formenty

**Affiliations:** 1 Integrated Research Facility at Fort Detrick, National Institute of Allergy and Infectious Diseases, National Institutes of Health, Fort Detrick, Frederick, MD 21702, USA; E-Mails: anna@logosconsulting.us (A.N.C.); jahrlingp@niaid.nih.gov (P.B.J.); wadaj@niaid.nih.gov (J.W.); 2 FAS Center for Systems Biology, Harvard University, Cambridge, MA 02138, USA; E-Mails: kandersen@oeb.harvard.edu (K.G.A.); sgire@oeb.harvard.edu (S.K.G.); pardis@broadinstitute.org (P.C.S.); nyozwiak@broadinstitute.org (N.L.Y.); 3 Unité de Biologie des Infections Virales Emergentes, Institut Pasteur, Lyon, France; Centre International de Recherche en Infectiologie (CIRI), Université de Lyon, INSERM U1111, Ecole Normale Supérieure de Lyon, Université Lyon 1, CNRS UMR5308, Lyon, France; E-Mail: sylvain.baize@inserm.fr; 4 Information Engineering Branch, National Center for Biotechnology Information, National Library of Medicine, National Institutes of Health, Bethesda, MD 20894, USA; E-Mails: bao@ncbi.nlm.nih.gov (Y.B.); olga.blinkova@nih.gov (O.B.); jamesbr@ncbi.nlm.nih.gov (J.R.B.); 5 United States Army Medical Research Institute of Infectious Diseases, Fort Detrick, Frederick, MD 21702, USA; E-Mails: sina.bavari.civ@mail.mil (S.B.).; jeffrey.r.kugelman.mil@mail.mil (J.R.K.); gustavo.f.palacios.ctr@us.army.mil (G.P.); sheli.r.radoshitzky.ctr@mail.mil (S.R.R.); cynthia.a.rossi.civ@mail.mil (C.A.R.); randal.j.schoepp.civ@mail.mil (R.J.S.); 6 Centre International de Recherches Médicales de Franceville, B. P. 769, Franceville, Gabon; E-Mails: nicolas.berthet@pasteur.fr (N.B.); eric.leroy@ird.fr (E.M.L.); gael_maganga@yahoo.fr (G.D.M.); 7 Fondation Mérieux, Washington, DC 20036, USA; E-Mail: joseph.fair@fondation-merieux.org; 8 Bernhard Nocht Institute for Tropical Medicine, World Health Organization (WHO) Collaborating Center for Arbovirus and Hemorrhagic Fever Reference and Research, and the German Center for Infection Research (DZIF), Partner Site Hamburg, 20259 Hamburg, Germany; E-Mails: gabriel@bni-hamburg.de (M.G.); guenther@bni.uni-hamburg.de (S.G.); 9 Tulane University School of Medicine, New Orleans, LA 70112, USA; E-Mails: rfgarry@tulane.edu (R.F.G.); lmoses2@tulane.edu (L.M.M.); jschieff@tulane.edu (J.S.S.); 10 Kenema Government Hospital, Kenema, Sierra Leone; E-Mail: augstgoba@yahoo.com; 11 Metabiota, Inc., San Francisco, CA 94104, USA; E-Mails: jpgonzalez@metabiota.com (J.-P.G.); nadia.wauquier@gmail.com (N.W.); 12 Department of Biological Sciences, College of Natural Sciences, and African Centre of Excellence for Genomics of Infectious Diseases, Redeemer’s University, Mowe, Ogun State, Nigeria; E-Mail: happic@run.edu.ng; 13 Institut National de Recherche Biomédicales, Kinshasa-Gombe BP 1197, Republic of the Congo; E-Mails: jimmy_kap@hotmail.com (J.K.); mbalaplacide@gmail.com (P.K.M.); muyembejj@gmail.com (J.-J.M.-T.); 14 Special Pathogens Program, National Microbiology Laboratory, Public Health Agency of Canada, Winnipeg, MB R3E 3R2, Canada; E-Mail: gary.kobinger@phac-aspc.gc.ca; 15 Université Gamal Abdel Nasser de Conakry, Laboratoire des fièvres hémorragiques en Guinée, Hôpital National Donka, Service des Maladies Infectieuses et Tropicales, BP 5680, Conakry, Guinea; E-Mail: cmagassouba01@gmail.com; 16 Viral Special Pathogens Branch, Division of High-Consequence Pathogens and Pathology, National Center for Emerging and Zoonotic Infectious Diseases, Centers for Disease Control and Prevention, Atlanta, GA 30333, USA; E-Mails: stn1@cdc.gov (S.T.N.); jit8@cdc.gov (J.S.T.); 17 Department of Medical Microbiology and Parasitology, College of Medicine of the University of Lagos, Idi-Araba, Private Mail Bag 12003, Lagos, Nigeria; E-Mail: omilabusa@yahoo.com; 18 The Broad Institute, Cambridge, MA 02142, USA; E-Mail: dpark@broadinstitute.org; 19 Center for Emerging and Zoonotic Diseases, National Institute for Communicable Diseases of the National Health Laboratory Service, Sandringham-Johannesburg 2192, Gauteng, South Africa; E-Mail: januszp@nicd.ac.za; 20 Computer Science and Artificial Intelligence Laboratory, Massachusetts Institute of Technology, Cambridge, MA 02139, USA; E-Mail: sealfon@gmail.com; 21 Zoonoses Research Unit, University of Pretoria, Private bag X20 Hatfield, Pretoria 0028, South Africa; E-Mail: bobswanepoel@gmail.com; 22 World Health Organization, 1211 Geneva, Switzerland; E-Mail: formentyp@who.int

**Keywords:** Ebola, Ebola virus, ebolavirus, filovirid, *Filoviridae*, filovirus, genome annotation, Lomela, Lokolia, Makona, mononegavirad, *Mononegavirales*, mononegavirus, virus classification, virus isolate, virus nomenclature, virus strain, virus taxonomy, virus variant

## Abstract

In 2014, Ebola virus (EBOV) was identified as the etiological agent of a large and still expanding outbreak of Ebola virus disease (EVD) in West Africa and a much more confined EVD outbreak in Middle Africa. Epidemiological and evolutionary analyses confirmed that all cases of both outbreaks are connected to a single introduction each of EBOV into human populations and that both outbreaks are not directly connected. Coding-complete genomic sequence analyses of isolates revealed that the two outbreaks were caused by two novel EBOV variants, and initial clinical observations suggest that neither of them should be considered strains. Here we present consensus decisions on naming for both variants (West Africa: “Makona”, Middle Africa: “Lomela”) and provide database-compatible full, shortened, and abbreviated names that are in line with recently established filovirus sub-species nomenclatures.

## 1. Introduction

On 10 March 2014, a viral hemorrhagic fever (VHF) outbreak was reported among humans in Guinea, West Africa [1]. Ebola virus (EBOV), the sole member of the species *Zaire ebolavirus* (genus *Ebolavirus*, family *Filoviridae*, order *Mononegavirales* [2]), was identified as the etiological agent. Consequently, the VHF was identified as Ebola virus disease (EVD) [1,3,4]. At the time of writing, this EVD outbreak has spread from Guinea into Liberia, Nigeria, Senegal, Sierra Leone, and Mali, with individual case exportations or transport of patients to France, Germany, Norway, Spain, UK, and US. At least 15351 human infections and 5459 deaths (proportion of fatal cases ≈36%) have been recorded as of 21 November 2014, making this outbreak the largest EVD outbreak in history [5]. Through conventional Sanger [1] and next-generation sequencing [6], 102 coding-complete EBOV genome sequences have been assembled (complete genome sequences with the exception of the ultimate 3’ and 5’ untranslated regions [7]) originating from three patients from Guinea and 78 patients from Sierra Leone [1,6]. Evolutionary analyses combined with epidemiological data demonstrate that all cases are directly epidemiologically linked, tracing back to a single introduction of EBOV into the human population [1,6] as has been found for most past EVD outbreaks [8].

On 24 August 2014, another EVD outbreak was reported from Boende District, Democratic Republic of the Congo, Middle Africa [9]. A total of 66 cases and 49 deaths (proportion of fatal case ≈74%) have been recorded [5]. As in Guinea, epidemiological analyses point towards a single introduction of EBOV from its unknown natural reservoir into the human population, with subsequent spread among humans by direct person-to-person transmission [9]. Thus far, two partial *L* (RNA-dependent RNA polymerase) gene sequences have been deposited into GenBank, and the coding-complete sequence of one isolate has been determined [9]. Phylogenetic analysis demonstrated that the Guinea and Democratic Republic of Congo EVD outbreaks were not related. The EBOV variants causing both outbreaks were distinct from each other and from variants known from previous EVD outbreaks [1,3,4,6,9].

Next-generation sequencing techniques enable the determination of coding-complete EBOV genomes in dozens and theoretically hundreds of clinical samples in parallel in the absence of classical virus culture [6]. The rapid accumulation of sequence data challenges sequence database curators and end users when novel sequences are not uniquely named according to common standards. Here, we assign final designations to the two EBOV variants causing the 2014 Guinea and Democratic Republic of Congo EVD outbreaks and update the current GenBank sequence entries accordingly.

## 2. Ebola Virus Strain, Variant, and Isolate Naming

In 2013, a consortium of filovirologists and sequence database experts working at the US National Center for Biotechnology Information (NCBI) established a consistent and prospective filovirus nomenclature below the species level [10]. This nomenclature, which already has been applied to all filovirus entries in NCBI’s RefSeq database [11], is based on the template:

<virus name>(/<strain>)/<isolation host-suffix>/<country of sampling>/<year of sampling>/<genetic variant designation>-<isolate designation>. [Note: the “/” between the <virus name> and the <isolation host-suffix> field was missing in the full name template outlined in [10], but is necessary for computational purposes. It is therefore introduced here and already implemented filovirus full names [11] will be retrospectively corrected.]

The <virus name> field should contain a filovirus name as outlined in [10]. Currently, the accepted filovirus names and abbreviations are Bundibugyo virus (BDBV), Ebola virus (EBOV), Lloviu virus (LLOV), Marburg virus (MARV), Ravn virus (RAVV), Reston virus (RESTV), Sudan virus (SUDV), and Taï Forest ebolavirus (TAFV), Table 1 [2,12].

**Table 1:** Summary of the current filovirus taxonomy endorsed by the 2012–2014 ICTV *Filoviridae* Study Group and accepted by the ICTV [2,12,13,14,15].

Current Taxonomy and Nomenclature (Ninth ICTV Report and Updates)
Order *Mononegavirales*
Family *Filoviridae*
Genus *Marburgvirus*
Species *Marburg marburgvirus*
Virus 1: Marburg virus (MARV)
Virus 2: Ravn virus (RAVV)
Genus *Ebolavirus*
Species *Taï Forest ebolavirus*
Virus: Taï Forest virus (TAFV)
Species *Reston ebolavirus*
Virus: Reston virus (RESTV)
Species *Sudan ebolavirus*
Virus: Sudan virus (SUDV)
Species *Zaire ebolavirus*
Virus: Ebola virus (EBOV)
Species *Bundibugyo ebolavirus*
Virus: Bundibugyo virus (BDBV)
Genus *Cuevavirus*
Species *Lloviu cuevavirus*
Virus: Lloviu virus (LLOV)

The <strain> field should contain a unique strain name in case the virus in question fulfills the criteria for being a strain (see [10]). The <isolation host-suffix> field should be provided in one word in the format “first letter of host genus name.full name of species descriptor” (e.g., “H.sapiens”) followed by suffix that denotes whether the sequence stems from an unpassaged sample (“-wt”), from virus isolated in tissue culture (“-tc”), or is a genomic fragment (“-frag). The <country of sampling> and <year of sampling> fields should contain the alpha-3 three-letter ISO 3166-1 code for the country where the virus was isolated and the year in which it was isolated, respectively. Finally, the <variant designation> and <isolate designation> fields should contain a unique variant name (*i.e.*, a name for the virus variant that was introduced into the human population that caused an outbreak) and unique isolate name (*i.e.*, the name for a particular representative of the variant), respectively [10]. To simplify manuscript writing, shortened and abbreviated virus designations are also defined [10]. For instance, the designations
full:Ebola virus/H.sapiens-tc/COD/1976/Yambuku-Ecranshortened:EBOV/H.sap/COD/76/Yam-Ecrabbreviated:EBOV/Yam-Ecr
specify an isolate “Ecran” of Ebola virus as a representative of the variant “Yambuku” (not a strain) that originated from a human in the Democratic Republic of the Congo in 1976 and was isolated/sequenced using tissue culture [16].

## 3. The 2014 Ebola Virus Variant Originating in Guinea

**Figure 1:**
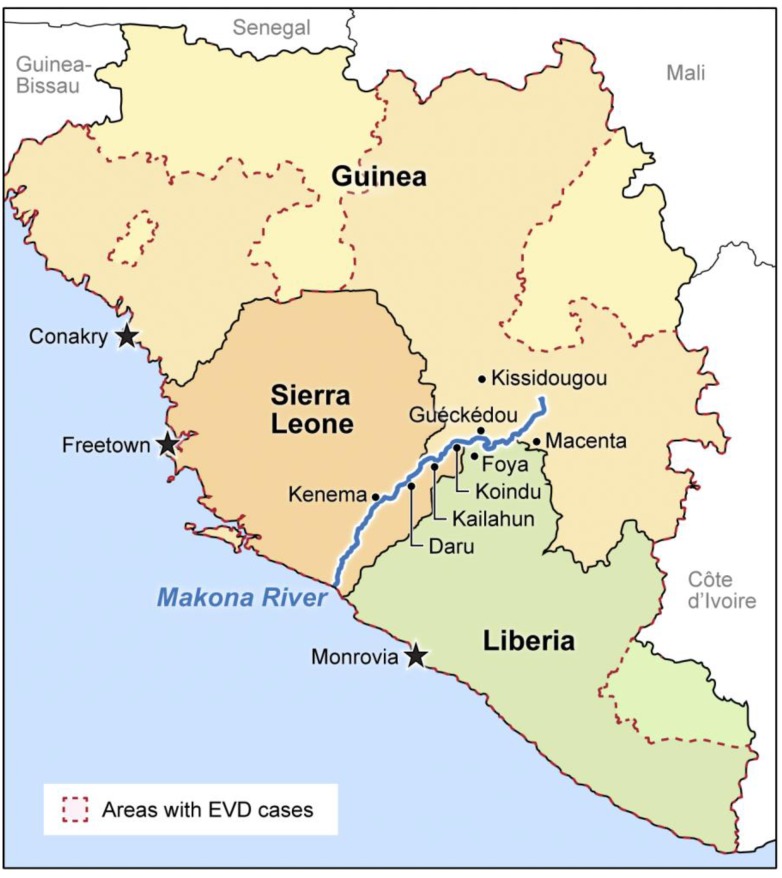
Location of the Makona River.

At the end of 2013, EVD broke out around Guéckédou, Kissidougou, and Macenta, Guinea [1] and consequently spread to at least five additional West African countries. Epidemiological and phylogenetic studies indicate that this large EVD outbreak was caused by a single introduction of one particular ebolavirus, Ebola virus (EBOV), into humans (*Homo sapiens*) from an unknown reservoir and therefore that all subsequent human cases (over 15,000 cases) are derived from one unnamed variant [1,6]. Preliminary clinical observations among EVD patients in West Africa do not contradict past descriptions of EVD [17,18,19,20,21,22], *i.e.*, this novel unnamed EBOV variant is not a strain as defined in standardized filovirus nomenclature [10]. Here we propose the name “Makona” (IPA: [mɑ'kɔnə] or [məˈkoʊnə]; English phonetic notation: mah-**kaw**-n*uh* or m*uh*-**koh**-n*uh*) after the Makona River close to the border between Liberia, Guinea, and Sierra Leone (Figure 1) as the variant name for this West African virus. The general name for the 2014 West African virus is therefore:

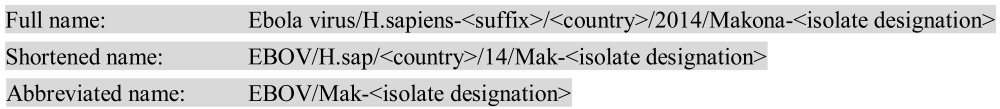


At the time of writing, 102 coding-complete genomic sequences of EBOV/Mak have been deposited into GenBank, all of which were obtained directly from clinical samples (“p0”) [1,6]. Following the rules laid out in filovirus standardized nomenclature [10], the names for these sequences therefore should contain the <suffix> “-wt”. In addition, one fragment of the L gene of one isolate of EBOV/Mak was deposited. Based on definitions described in standardized nomenclature [10], the corresponding <suffix> field should therefore be filled with “-frag”. All currently deposited sequences stem from either Guinean, Sierra Leonean, or Nigerian samples. The 3-letter country codes to be used the <country> field [10] for all countries that have thus far handled patients infected with EBOV/Mak are summarized in Table 2.

**Table 2:** ISO 3166-1 alpha-3 country codes for countries with recorded cases connected to the 2014 EVD epidemic that started in Guinea [23]:

Official short country name in English (geographical name)	Official country name in English (protocol name)	ISO 3166-1 3-letter abbreviation
France	French Republic	FRA
Germany	Federal Republic of Germany	DEU
Guinea	Republic of Guinea	GIN
LiberiaMaliNigeria	Republic of LiberiaRepublic of MaliFederal Republic of Nigeria	LBRMLINGA
NorwaySenegal	Kingdom of NorwayRepublic of Senegal	NORSEN
Sierra Leone	Republic of Sierra Leone	SLE
Spain	Kingdom of Spain	ESP
United Kingdom	United Kingdom of Great Britain and Northern Ireland	GBR
United States	United States of America	USA

Three replicating isolates (C05, C07, and C15) of EBOV/Mak have been reported [1]. The 102 coding-complete genomes (including those of C05, C07, and C15) and the one fragmented L gene sequence that have been deposited into GenBank all already have assigned unique <isolate designation> descriptors. Accordingly, in all currently deposited sequences of EBOV/Mak, the definition line will be adjusted to “*Zaire ebolavirus* isolate Ebola virus/H.sapiens-<suffix>/<country>/2014/Makona-<isolate designation>, [coding-]complete genome, with the <suffix>, <country>, and <isolate designation> fields will be filled according to their origin. The GenBank <strain> field will be cleared throughout; the Genbank <isolate> field will be filled with “Ebola virus/H.sapiens-<suffix>/<country>/2014/Makona-<isolate designation>”, and the <organism> field will be corrected, if necessary, to “*Zaire ebolavirus*” (Table 3).

**Table 3:** EBOV isolates from the West African EVD outbreak, 2014: Updated and/or corrected GenBank fields and final names

GenBank Accession Number	<GenBank field> = Updated/corrected information

	Final names
KJ660346	<DEFINITION LINE> *= Zaire ebolavirus*^1^ isolate Ebola virus/H.sapiens-wt/GIN/2014/Makona-C15, [coding-]complete genome^2^
	<SOURCE ORGANISM> *= Zaire ebolavirus*^1^
	</isolate> = Ebola virus/H.sapiens-wt/GIN/2014/Makona-C15
	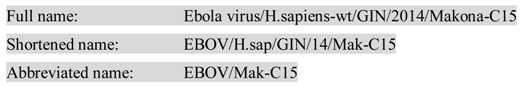
KJ660347	<DEFINITION LINE> *= Zaire ebolavirus*^1^ isolate Ebola virus/H.sapiens-wt/GIN/2014/Makona-C07, [coding-]complete genome^2^
	<SOURCE ORGANISM> *= Zaire ebolavirus*^1^
	</isolate> = Ebola virus/H.sapiens-wt/GIN/2014/Makona-C07
	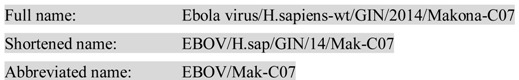
KJ660348	<DEFINITION LINE> *= Zaire ebolavirus*^1^ isolate Ebola virus/H.sapiens-wt/GIN/2014/Makona-C05, [coding-]complete genome^2^
	<SOURCE ORGANISM> *= Zaire ebolavirus*^1^
	</isolate> = Ebola virus/H.sapiens-wt/GIN/2014/Makona-C05
	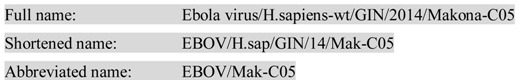
KM034549	<DEFINITION LINE> *= Zaire ebolavirus*^1^ isolate Ebola virus/H.sapiens-wt/SLE/2014/Makona-EM095B, [coding-]complete genome^2^
	<SOURCE ORGANISM> *= Zaire ebolavirus*^1^
	</isolate> = Ebola virus/H.sapiens-wt/SLE/2014/Makona-EM095B
	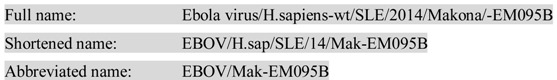
KM034550	<DEFINITION LINE> *= Zaire ebolavirus*^1^ isolate Ebola virus/H.sapiens-wt/SLE/2014/Makona-EM095, [coding-]complete genome^2^
	<SOURCE ORGANISM> *= Zaire ebolavirus*^1^
	</isolate> = Ebola virus/H.sapiens-wt/SLE/2014/Makona-EM095
	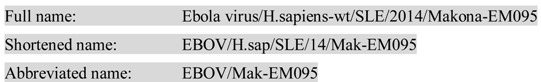
KM034551	<DEFINITION LINE> *= Zaire ebolavirus*^1^ isolate Ebola virus/H.sapiens-wt/SLE/2014/Makona-EM096, [coding-]complete genome^2^
	<SOURCE ORGANISM> *= Zaire ebolavirus*^1^
	</isolate> = Ebola virus/H.sapiens-wt/SLE/2014/Makona-EM096
	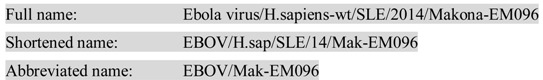
KM034552	<DEFINITION LINE> *= Zaire ebolavirus*^1^ isolate Ebola virus/H.sapiens-wt/SLE/2014/Makona-EM098, [coding-]complete genome^2^
	<SOURCE ORGANISM> *= Zaire ebolavirus*^1^
	</isolate> = Ebola virus/H.sapiens-wt/SLE/2014/Makona-EM098
	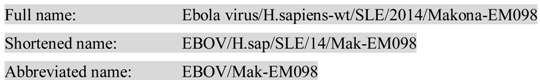
KM034553	<DEFINITION LINE> *= Zaire ebolavirus*^1^ isolate Ebola virus/H.sapiens-wt/SLE/2014/Makona-G3670.1, [coding-]complete genome^2^
	<SOURCE ORGANISM> *= Zaire ebolavirus*^1^
	</isolate> = Ebola virus/H.sapiens-wt/SLE/2014/Makona-G3670.1
	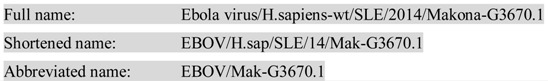
KM034554	<DEFINITION LINE> *= Zaire ebolavirus*^1^ isolate Ebola virus/H.sapiens-wt/SLE/2014/Makona-G3676.1, [coding-]complete genome^2^
	<SOURCE ORGANISM> *= Zaire ebolavirus*^1^
	</isolate> = Ebola virus/H.sapiens-wt/SLE/2014/Makona-G3676.1
	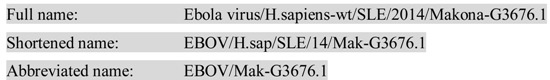
KM034555	<DEFINITION LINE> *= Zaire ebolavirus*^1^ isolate Ebola virus/H.sapiens-wt/SLE/2014/Makona-G3676.2, [coding-]complete genome^2^
	<SOURCE ORGANISM> *= Zaire ebolavirus*^1^
	</isolate> = Ebola virus/H.sapiens-wt/SLE/2014/Makona-G3676.2
	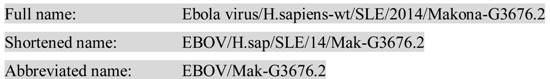
KM034556	<DEFINITION LINE> *= Zaire ebolavirus*^1^ isolate Ebola virus/H.sapiens-wt/SLE/2014/Makona-G3677.1, [coding-]complete genome^2^
	<SOURCE ORGANISM> *= Zaire ebolavirus*^1^
	</isolate> = Ebola virus/H.sapiens-wt/SLE/2014/Makona-G3677.1
	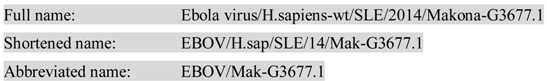
KM034557	<DEFINITION LINE> *= Zaire ebolavirus*^1^ isolate Ebola virus/H.sapiens-wt/SLE/2014/Makona-G3677.2, [coding-]complete genome^2^
	<SOURCE ORGANISM> *= Zaire ebolavirus*^1^
	</isolate> = Ebola virus/H.sapiens-wt/SLE/2014/Makona-G3677.2
	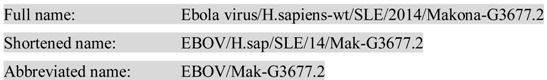
KM034558	<DEFINITION LINE> *= Zaire ebolavirus*^1^ isolate Ebola virus/H.sapiens-wt/SLE/2014/Makona-G3679.1, [coding-]complete genome^2^
	<SOURCE ORGANISM> *= Zaire ebolavirus*^1^
	</isolate> = Ebola virus/H.sapiens-wt/SLE/2014/Makona-G3679.1
	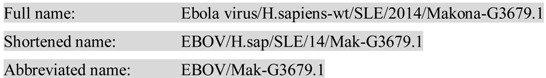
KM034559	<DEFINITION LINE> *= Zaire ebolavirus*^1^ isolate Ebola virus/H.sapiens-wt/SLE/2014/Makona-G3680.1, [coding-]complete genome^2^
	<SOURCE ORGANISM> *= Zaire ebolavirus*^1^
	</isolate> = Ebola virus/H.sapiens-wt/SLE/2014/Makona-G3680.1
	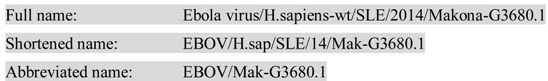
KM034560	<DEFINITION LINE> *= Zaire ebolavirus*^1^ isolate Ebola virus/H.sapiens-wt/SLE/2014/Makona-G3682.1, [coding-]complete genome^2^
	<SOURCE ORGANISM> *= Zaire ebolavirus*^1^
	</isolate> = Ebola virus/H.sapiens-wt/SLE/2014/Makona-G3682.1
	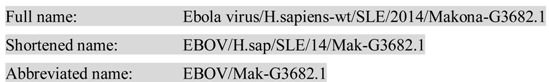
KM034561	<DEFINITION LINE> *= Zaire ebolavirus*^1^ isolate Ebola virus/H.sapiens-wt/SLE/2014/Mak-G3683.1, [coding-]complete genome^2^
	<SOURCE ORGANISM> *= Zaire ebolavirus*^1^
	</isolate> = Ebola virus/H.sapiens-wt/SLE/2014/Mak-G3683.1
	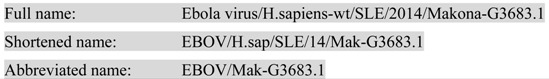
KM034562	<DEFINITION LINE> *= Zaire ebolavirus*^1^ isolate Ebola virus/H.sapiens-wt/SLE/2014/Mak-G3686.1, [coding-]complete genome^2^
	<SOURCE ORGANISM> *= Zaire ebolavirus*^1^
	</isolate> = Ebola virus/H.sapiens-wt/SLE/2014/Makona-G3686.1
	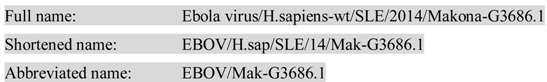
KM034563	<DEFINITION LINE> *= Zaire ebolavirus*^1^ isolate Ebola virus/H.sapiens-wt/SLE/2014/Makona-G3687.1, [coding-]complete genome^2^
	<SOURCE ORGANISM> *= Zaire ebolavirus*^1^
	</isolate> = Ebola virus/H.sapiens-wt/SLE/2014/Makona-G3687.1
	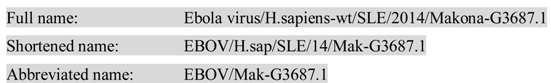
KM233035	<DEFINITION LINE> *= Zaire ebolavirus*^1^ isolate Ebola virus/H.sapiens-wt/SLE/2014/Makona-EM104, [coding-]complete genome^2^
	<SOURCE ORGANISM> *= Zaire ebolavirus*^1^
	</isolate> = Ebola virus/H.sapiens-wt/SLE/2014/Makona-EM104
	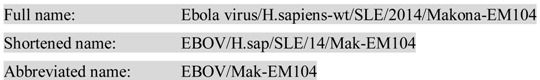
KM233036	<DEFINITION LINE> *= Zaire ebolavirus*^1^ isolate Ebola virus/H.sapiens-wt/SLE/2014/Makona-EM106, [coding-]complete genome^2^
	<SOURCE ORGANISM> *= Zaire ebolavirus*^1^
	</isolate> = Ebola virus/H.sapiens-wt/SLE/2014/Makona-EM106
	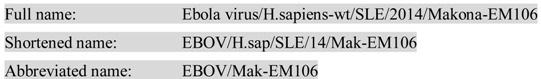
KM233037	<DEFINITION LINE> *= Zaire ebolavirus*^1^ isolate Ebola virus/H.sapiens-wt/SLE/2014/Makona-EM110, [coding-]complete genome^2^
	<SOURCE ORGANISM> *= Zaire ebolavirus*^1^
	</isolate> = Ebola virus/H.sapiens-wt/SLE/2014/Makona-EM110
	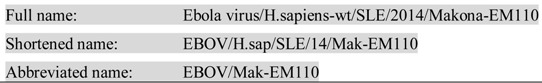
KM233038	<DEFINITION LINE> *= Zaire ebolavirus*^1^ isolate Ebola virus/H.sapiens-wt/SLE/2014/Makona-EM111, [coding-]complete genome^2^
	<SOURCE ORGANISM> *= Zaire ebolavirus*^1^
	</isolate> = Ebola virus/H.sapiens-wt/SLE/2014/Makona-EM111
	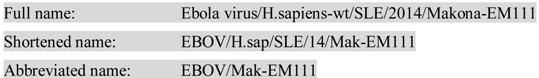
KM233039	<DEFINITION LINE> *= Zaire ebolavirus*^1^ isolate Ebola virus/H.sapiens-wt/SLE/2014/Makona-EM112, [coding-]complete genome^2^
	<SOURCE ORGANISM> *= Zaire ebolavirus*^1^
	</isolate> = Ebola virus/H.sapiens-wt/SLE/2014/Makona-EM112
	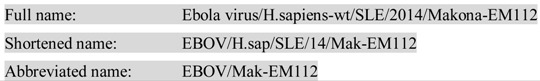
KM233040	<DEFINITION LINE> *= Zaire ebolavirus*^1^ isolate Ebola virus/H.sapiens-wt/SLE/2014/Makona-EM113, [coding-]complete genome^2^
	<SOURCE ORGANISM> *= Zaire ebolavirus*^1^
	</isolate> = Ebola virus/H.sapiens-wt/SLE/2014/Makona-EM113
	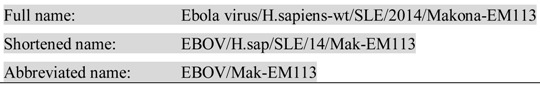
KM233041	<DEFINITION LINE> *= Zaire ebolavirus*^1^ isolate Ebola virus/H.sapiens-wt/SLE/2014/Makona-EM115, [coding-]complete genome^2^
	<SOURCE ORGANISM> *= Zaire ebolavirus*^1^
	</isolate> = Ebola virus/H.sapiens-wt/SLE/2014/Makona-EM115
	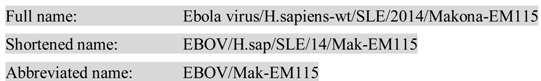
KM233042	<DEFINITION LINE> *= Zaire ebolavirus*^1^ isolate Ebola virus/H.sapiens-wt/SLE/2014/Makona-EM119, [coding-]complete genome^2^
	<SOURCE ORGANISM> *= Zaire ebolavirus*^1^
	</isolate> = Ebola virus/H.sapiens-wt/SLE/2014/Makona-EM119
	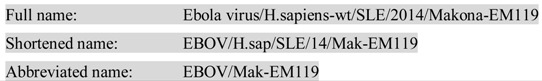
KM233043	<DEFINITION LINE> *= Zaire ebolavirus*^1^ isolate Ebola virus/H.sapiens-wt/SLE/2014/Makona-EM120, [coding-]complete genome^2^
	<SOURCE ORGANISM> *= Zaire ebolavirus*^1^
	</isolate> = Ebola virus/H.sapiens-wt/SLE/2014/Makona-EM120
	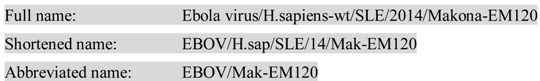
KM233044	<DEFINITION LINE> *= Zaire ebolavirus*^1^ isolate Ebola virus/H.sapiens-wt/SLE/2014/Makona-EM121, [coding-]complete genome^2^
	<SOURCE ORGANISM> *= Zaire ebolavirus*^1^
	</isolate> = Ebola virus/H.sapiens-wt/SLE/2014/Makona-EM121
	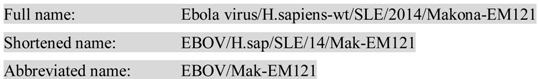
KM233045	<DEFINITION LINE> *= Zaire ebolavirus*^1^ isolate Ebola virus/H.sapiens-wt/SLE/2014/Makona-EM124.1, [coding-]complete genome^2^
	<SOURCE ORGANISM> *= Zaire ebolavirus*^1^
	</isolate> = Ebola virus/H.sapiens-wt/SLE/2014/Makona-EM124.1
	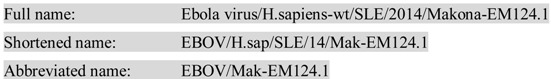
KM233046	<DEFINITION LINE> *= Zaire ebolavirus*^1^ isolate Ebola virus/H.sapiens-wt/SLE/2014/Makona-EM124.2, [coding-]complete genome^2^
	<SOURCE ORGANISM> *= Zaire ebolavirus*^1^
	</isolate> = Ebola virus/H.sapiens-wt/SLE/2014/Makona-EM124.2
	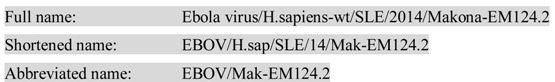
KM233047	<DEFINITION LINE> *= Zaire ebolavirus*^1^ isolate Ebola virus/H.sapiens-wt/SLE/2014/Makona-EM124.3, [coding-]complete genome^2^
	<SOURCE ORGANISM> *= Zaire ebolavirus*^1^
	</isolate> = Ebola virus/H.sapiens-wt/SLE/2014/Makona-EM124.3
	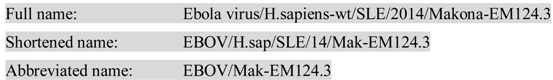
KM233048	<DEFINITION LINE> *= Zaire ebolavirus*^1^ isolate Ebola virus/H.sapiens-wt/SLE/2014/Makona-EM124.4, [coding-]complete genome^2^
	<SOURCE ORGANISM> *= Zaire ebolavirus*^1^
	</isolate> = Ebola virus/H.sapiens-wt/SLE/2014/Makona-EM124.4
	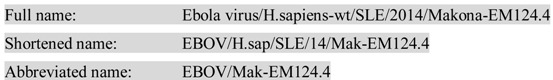
KM233049	<DEFINITION LINE> *= Zaire ebolavirus*^1^ isolate Ebola virus/H.sapiens-wt/SLE/2014/Makona-G3707, [coding-]complete genome^2^
	<SOURCE ORGANISM> *= Zaire ebolavirus*^1^
	</isolate> = Ebola virus/H.sapiens-wt/SLE/2014/Makona-G3707
	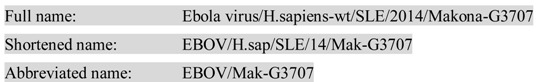
KM233050	<DEFINITION LINE> *= Zaire ebolavirus*^1^ isolate Ebola virus/H.sapiens-wt/SLE/2014/Makona-G3713.2, [coding-]complete genome^2^
	<SOURCE ORGANISM> *= Zaire ebolavirus*^1^
	</isolate> = Ebola virus/H.sapiens-wt/SLE/2014/Makona-G3713.2
	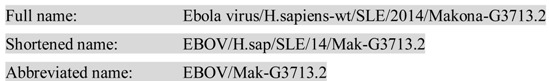
KM233051	<DEFINITION LINE> *= Zaire ebolavirus*^1^ isolate Ebola virus/H.sapiens-wt/SLE/2014/Makona-G3713.3, [coding-]complete genome^2^
	<SOURCE ORGANISM> *= Zaire ebolavirus*^1^
	</isolate> = Ebola virus/H.sapiens-wt/SLE/2014/Makona-G3713.3
	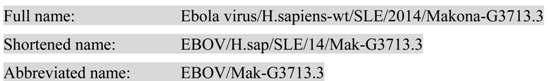
KM233052	<DEFINITION LINE> *= Zaire ebolavirus*^1^ isolate Ebola virus/H.sapiens-wt/SLE/2014/Makona-G3713.4, [coding-]complete genome^2^
	<SOURCE ORGANISM> *= Zaire ebolavirus*^1^
	</isolate> = Ebola virus/H.sapiens-wt/SLE/2014/Makona-G3713.4
	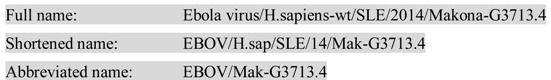
KM233053	<DEFINITION LINE> *= Zaire ebolavirus*^1^ isolate Ebola virus/H.sapiens-wt/SLE/2014/Makona-G3724, [coding-]complete genome^2^
	<SOURCE ORGANISM> *= Zaire ebolavirus*^1^
	</isolate> = Ebola virus/H.sapiens-wt/SLE/2014/Makona-G3724
	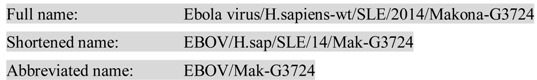
KM233054	<DEFINITION LINE> *= Zaire ebolavirus*^1^ isolate Ebola virus/H.sapiens-wt/SLE/2014/Makona-G3729, [coding-]complete genome^2^
	<SOURCE ORGANISM> *= Zaire ebolavirus*^1^
	</isolate> = Ebola virus/H.sapiens-wt/SLE/2014/Makona-G3729
	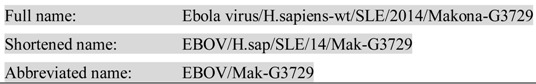
KM233055	<DEFINITION LINE> *= Zaire ebolavirus*^1^ isolate Ebola virus/H.sapiens-wt/SLE/2014/Makona-G3734.1, [coding-]complete genome^2^
	<SOURCE ORGANISM> *= Zaire ebolavirus*^1^
	</isolate> = Ebola virus/H.sapiens-wt/SLE/2014/Makona-G3734.1
	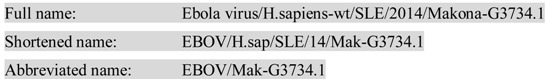
KM233056	<DEFINITION LINE> *= Zaire ebolavirus*^1^ isolate Ebola virus/H.sapiens-wt/SLE/2014/Makona-G3735.1, [coding-]complete genome^2^
	<SOURCE ORGANISM> *= Zaire ebolavirus*^1^
	</isolate> = Ebola virus/H.sapiens-wt/SLE/2014/Makona-G3735.1
	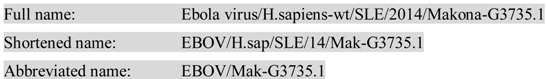
KM233057	<DEFINITION LINE> *= Zaire ebolavirus*^1^ isolate Ebola virus/H.sapiens-wt/SLE/2014/Makona-G3735.2, [coding-]complete genome^2^
	<SOURCE ORGANISM> *= Zaire ebolavirus*^1^
	</isolate> = Ebola virus/H.sapiens-wt/SLE/2014/Makona-G3735.2
	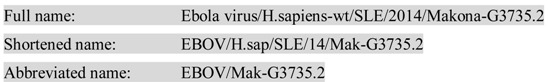
KM233058	<DEFINITION LINE> *= Zaire ebolavirus*^1^ isolate Ebola virus/H.sapiens-wt/SLE/2014/Makona-G3750.1, [coding-]complete genome^2^
	<SOURCE ORGANISM> *= Zaire ebolavirus*^1^
	</isolate> = Ebola virus/H.sapiens-wt/SLE/2014/Makona-G3750.1
	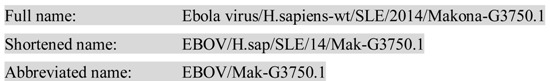
KM233059	<DEFINITION LINE> *= Zaire ebolavirus*^1^ isolate Ebola virus/H.sapiens-wt/SLE/2014/Makona-G3750.2, [coding-]complete genome^2^
	<SOURCE ORGANISM> *= Zaire ebolavirus*^1^
	</isolate> = Ebola virus/H.sapiens-wt/SLE/2014/Makona-G3750.2
	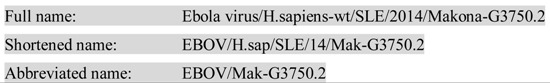
KM233060	<DEFINITION LINE> *= Zaire ebolavirus*^1^ isolate Ebola virus/H.sapiens-wt/SLE/2014/Makona-G3750.3, [coding-]complete genome^2^
	<SOURCE ORGANISM> *= Zaire ebolavirus*^1^
	</isolate> = Ebola virus/H.sapiens-wt/SLE/2014/Makona-G3750.3
	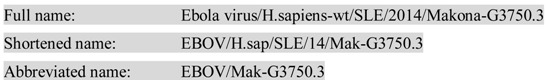
KM233061	<DEFINITION LINE> *= Zaire ebolavirus*^1^ isolate Ebola virus/H.sapiens-wt/SLE/2014/Makona-G3752, [coding-]complete genome^2^
	<SOURCE ORGANISM> *= Zaire ebolavirus*^1^
	</isolate> = Ebola virus/H.sapiens-wt/SLE/2014/Makona-G3752
	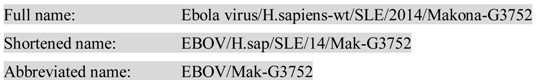
KM233062	<DEFINITION LINE> *= Zaire ebolavirus*^1^ isolate Ebola virus/H.sapiens-wt/SLE/2014/Makona-G3758, [coding-]complete genome^2^
	<SOURCE ORGANISM> *= Zaire ebolavirus*^1^
	</isolate> = Ebola virus/H.sapiens-wt/SLE/2014/Makona-G3758
	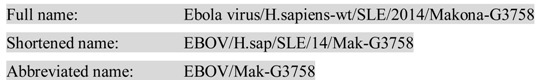
KM233063	<DEFINITION LINE> *= Zaire ebolavirus*^1^ isolate Ebola virus/H.sapiens-wt/SLE/2014/Makona-G3764, [coding-]complete genome^2^
	<SOURCE ORGANISM> *= Zaire ebolavirus*^1^
	</isolate> = Ebola virus/H.sapiens-wt/SLE/2014/Makona-G3764
	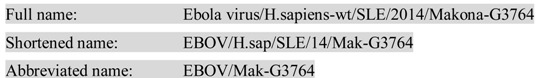
KM233064	<DEFINITION LINE> *= Zaire ebolavirus*^1^ isolate Ebola virus/H.sapiens-wt/SLE/2014/Makona-G3765.2, [coding-]complete genome^2^
	<SOURCE ORGANISM> *= Zaire ebolavirus*^1^
	</isolate> = Ebola virus/H.sapiens-wt/SLE/2014/Makona-G3765.2
	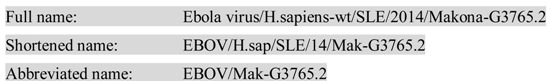
KM233065	<DEFINITION LINE> *= Zaire ebolavirus*^1^ isolate Ebola virus/H.sapiens-wt/SLE/2014/Makona-G3769.1, [coding-]complete genome^2^
	<SOURCE ORGANISM> *= Zaire ebolavirus*^1^
	</isolate> = Ebola virus/H.sapiens-wt/SLE/2014/Makona-G3769.1
	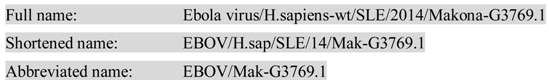
KM233066	<DEFINITION LINE> *= Zaire ebolavirus*^1^ isolate Ebola virus/H.sapiens-wt/SLE/2014/Makona-G3769.2, [coding-]complete genome^2^
	<SOURCE ORGANISM> *= Zaire ebolavirus*^1^
	</isolate> = Ebola virus/H.sapiens-wt/SLE/2014/Makona-G3769.2
	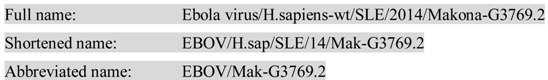
KM233067	<DEFINITION LINE> *= Zaire ebolavirus*^1^ isolate Ebola virus/H.sapiens-wt/SLE/2014/Makona-G3769.3, [coding-]complete genome^2^
	<SOURCE ORGANISM> *= Zaire ebolavirus*^1^
	</isolate> = Ebola virus/H.sapiens-wt/SLE/2014/Makona-G3769.3
	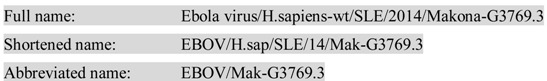
KM233068	<DEFINITION LINE> *= Zaire ebolavirus*^1^ isolate Ebola virus/H.sapiens-wt/SLE/2014/Makona-G3769.4, [coding-]complete genome^2^
	<SOURCE ORGANISM> *= Zaire ebolavirus*^1^
	</isolate> = Ebola virus/H.sapiens-wt/SLE/2014/Makona-G3769.4
	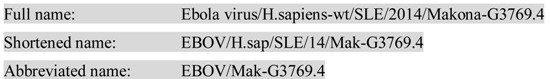
KM233069	<DEFINITION LINE> *= Zaire ebolavirus*^1^ isolate Ebola virus/H.sapiens-wt/SLE/2014/Makona-G3770.1, [coding-]complete genome^2^
	<SOURCE ORGANISM> *= Zaire ebolavirus*^1^
	</isolate> = Ebola virus/H.sapiens-wt/SLE/2014/Makona-G3770.1
	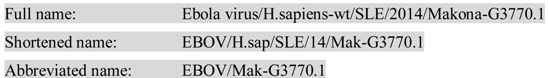
KM233070	<DEFINITION LINE> *= Zaire ebolavirus*^1^ isolate Ebola virus/H.sapiens-wt/SLE/2014/Makona-G3770.2, [coding-]complete genome^2^
	<SOURCE ORGANISM> *= Zaire ebolavirus*^1^
	</isolate> = Ebola virus/H.sapiens-wt/SLE/2014/Makona-G3770.2
	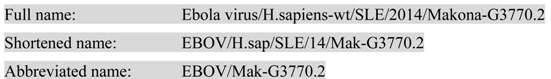
KM233071	<DEFINITION LINE> *= Zaire ebolavirus*^1^ isolate Ebola virus/H.sapiens-wt/SLE/2014/Makona-G3771, [coding-]complete genome^2^
	<SOURCE ORGANISM> *= Zaire ebolavirus*^1^
	</isolate> = Ebola virus/H.sapiens-wt/SLE/2014/Makona-G3771
	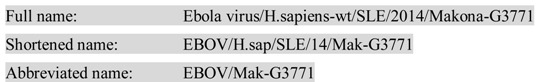
KM233072	<DEFINITION LINE> *= Zaire ebolavirus*^1^ isolate Ebola virus/H.sapiens-wt/SLE/2014/Makona-G3782, [coding-]complete genome^2^
	<SOURCE ORGANISM> *= Zaire ebolavirus*^1^
	</isolate> = Ebola virus/H.sapiens-wt/SLE/2014/Makona-G3782
	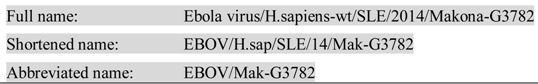
KM233073	<DEFINITION LINE> *= Zaire ebolavirus*^1^ isolate Ebola virus/H.sapiens-wt/SLE/2014/Makona-G3786, [coding-]complete genome^2^
	<SOURCE ORGANISM> *= Zaire ebolavirus*^1^
	</isolate> = Ebola virus/H.sapiens-wt/SLE/2014/Makona-G3786
	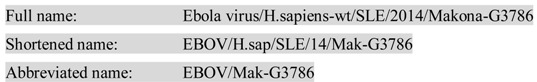
KM233074	<DEFINITION LINE> *= Zaire ebolavirus*^1^ isolate Ebola virus/H.sapiens-wt/SLE/2014/Makona-G3787, [coding-]complete genome^2^
	<SOURCE ORGANISM> *= Zaire ebolavirus*^1^
	</isolate> = Ebola virus/H.sapiens-wt/SLE/2014/Makona-G3787
	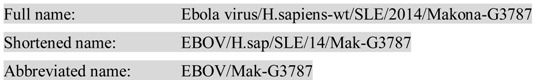
KM233075	<DEFINITION LINE> *= Zaire ebolavirus*^1^ isolate Ebola virus/H.sapiens-wt/SLE/2014/Makona-G3788, [coding-]complete genome^2^
	<SOURCE ORGANISM> *= Zaire ebolavirus*^1^
	</isolate> = Ebola virus/H.sapiens-wt/SLE/2014/Makona-G3788
	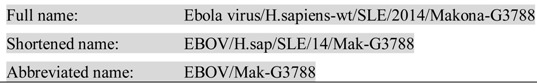
KM233076	<DEFINITION LINE> *= Zaire ebolavirus*^1^ isolate Ebola virus/H.sapiens-wt/SLE/2014/Makona-G3789.1, [coding-]complete genome^2^
	<SOURCE ORGANISM> *= Zaire ebolavirus*^1^
	</isolate> = Ebola virus/H.sapiens-wt/SLE/2014/Makona-G3789.1
	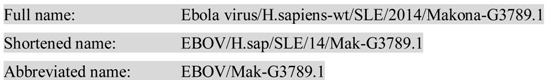
KM233077	<DEFINITION LINE> *= Zaire ebolavirus*^1^ isolate Ebola virus/H.sapiens-wt/SLE/2014/Makona-G3795, [coding-]complete genome^2^
	<SOURCE ORGANISM> *= Zaire ebolavirus*^1^
	</isolate> = Ebola virus/H.sapiens-wt/SLE/2014/Makona-G3795
	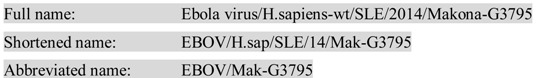
KM233078	<DEFINITION LINE> *= Zaire ebolavirus*^1^ isolate Ebola virus/H.sapiens-wt/SLE/2014/Makona-G3796, [coding-]complete genome^2^
	<SOURCE ORGANISM> *= Zaire ebolavirus*^1^
	</isolate> = Ebola virus/H.sapiens-wt/SLE/2014/Makona-G3796
	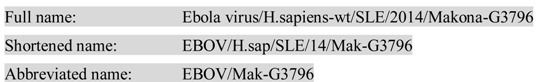
KM233079	<DEFINITION LINE> *= Zaire ebolavirus*^1^ isolate Ebola virus/H.sapiens-wt/SLE/2014/Makona-G3798, [coding-]complete genome^2^
	<SOURCE ORGANISM> *= Zaire ebolavirus*^1^
	</isolate> = Ebola virus/H.sapiens-wt/SLE/2014/Makona-G3798
	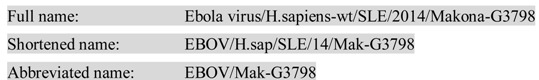
KM233080	<DEFINITION LINE> *= Zaire ebolavirus*^1^ isolate Ebola virus/H.sapiens-wt/SLE/2014/Makona-G3799, [coding-]complete genome^2^
	<SOURCE ORGANISM> *= Zaire ebolavirus*^1^
	</isolate> = Ebola virus/H.sapiens-wt/SLE/2014/Makona-G3799
	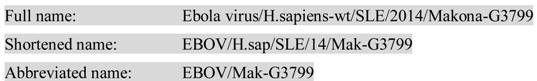
KM233081	<DEFINITION LINE> *= Zaire ebolavirus*^1^ isolate Ebola virus/H.sapiens-wt/SLE/2014/Makona-G3800, [coding-]complete genome^2^
	<SOURCE ORGANISM> *= Zaire ebolavirus*^1^
	</isolate> = Ebola virus/H.sapiens-wt/SLE/2014/Makona-G3800
	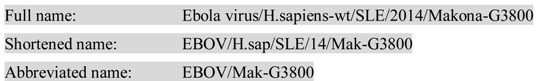
KM233082	<DEFINITION LINE> *= Zaire ebolavirus*^1^ isolate Ebola virus/H.sapiens-wt/SLE/2014/Makona-G3805.1, [coding-]complete genome^2^
	<SOURCE ORGANISM> *= Zaire ebolavirus*^1^
	</isolate> = Ebola virus/H.sapiens-wt/SLE/2014/Makona-G3805.1
	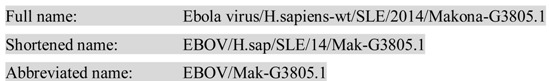
KM233083	<DEFINITION LINE> *= Zaire ebolavirus*^1^ isolate Ebola virus/H.sapiens-wt/SLE/2014/Makona-G3805.2, [coding-]complete genome^2^
	<SOURCE ORGANISM> *= Zaire ebolavirus*^1^
	</isolate> = Ebola virus/H.sapiens-wt/SLE/2014/Makona-G3805.2
	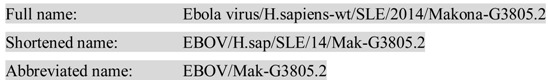
KM233084	<DEFINITION LINE> *= Zaire ebolavirus*^1^ isolate Ebola virus/H.sapiens-wt/SLE/2014/Makona-G3807, [coding-]complete genome^2^
	<SOURCE ORGANISM> *= Zaire ebolavirus*^1^
	</isolate> = Ebola virus/H.sapiens-wt/SLE/2014/Makona-G3807
	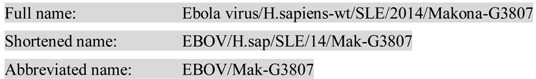
KM233085	<DEFINITION LINE> *= Zaire ebolavirus*^1^ isolate Ebola virus/H.sapiens-wt/SLE/2014/Makona-G3808, [coding-]complete genome^2^
	<SOURCE ORGANISM> *= Zaire ebolavirus*^1^
	</isolate> = Ebola virus/H.sapiens-wt/SLE/2014/Makona-G3808
	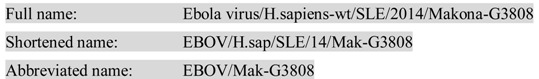
KM233086	<DEFINITION LINE> *= Zaire ebolavirus*^1^ isolate Ebola virus/H.sapiens-wt/SLE/2014/Makona-G3809, [coding-]complete genome^2^
	<SOURCE ORGANISM> *= Zaire ebolavirus*^1^
	</isolate> = Ebola virus/H.sapiens-wt/SLE/2014/Makona-G3809
	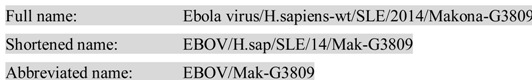
KM233087	<DEFINITION LINE> *= Zaire ebolavirus*^1^ isolate Ebola virus/H.sapiens-wt/SLE/2014/Makona-G3810.1, [coding-]complete genome^2^
	<SOURCE ORGANISM> *= Zaire ebolavirus*^1^
	</isolate> = Ebola virus/H.sapiens-wt/SLE/2014/Makona-G3810.1
	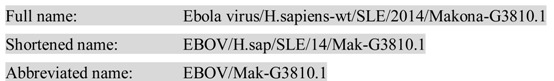
KM233088	<DEFINITION LINE> *= Zaire ebolavirus*^1^ isolate Ebola virus/H.sapiens-wt/SLE/2014/Makona-G3810.2, [coding-]complete genome^2^
	<SOURCE ORGANISM> *= Zaire ebolavirus*^1^
	</isolate> = Ebola virus/H.sapiens-wt/SLE/2014/Makona-G3810.2
	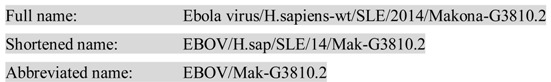
KM233089	<DEFINITION LINE> *= Zaire ebolavirus*^1^ isolate Ebola virus/H.sapiens-wt/SLE/2014/Makona-G3814, [coding-]complete genome^2^
	<SOURCE ORGANISM> *= Zaire ebolavirus*^1^
	</isolate> = Ebola virus/H.sapiens-wt/SLE/2014/Makona-G3814
	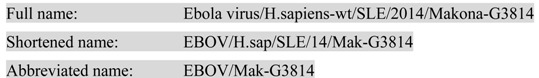
KM233090	<DEFINITION LINE> *= Zaire ebolavirus*^1^ isolate Ebola virus/H.sapiens-wt/SLE/2014/Makona-G3816, [coding-]complete genome^2^ *= Zaire ebolavirus*^1^
	</isolate> = Ebola virus/H.sapiens-wt/SLE/2014/Makona-G3816
	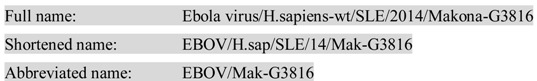
KM233091	<DEFINITION LINE> *= Zaire ebolavirus*^1^ isolate Ebola virus/H.sapiens-wt/SLE/2014/Makona-G3817, [coding-]complete genome^2^
	<SOURCE ORGANISM> *= Zaire ebolavirus*^1^
	</isolate> = Ebola virus/H.sapiens-wt/SLE/2014/Makona-G3817
	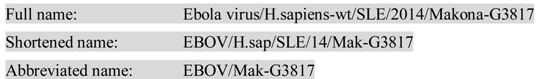
KM233092	<DEFINITION LINE> *= Zaire ebolavirus*^1^ isolate Ebola virus/H.sapiens-wt/SLE/2014/Makona-G3818, [coding-]complete genome^2^
	<SOURCE ORGANISM> *= Zaire ebolavirus*^1^
	</isolate> = Ebola virus/H.sapiens-wt/SLE/2014/Makona-G3818
	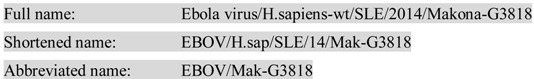
KM233093	<DEFINITION LINE> *= Zaire ebolavirus*^1^ isolate Ebola virus/H.sapiens-wt/SLE/2014/Makona-G3819, [coding-]complete genome^2^
	<SOURCE ORGANISM> *= Zaire ebolavirus*^1^
	</isolate> = Ebola virus/H.sapiens-wt/SLE/2014/Makona-G3819
	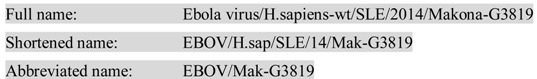
KM233094	<DEFINITION LINE> *= Zaire ebolavirus*^1^ isolate Ebola virus/H.sapiens-wt/SLE/2014/Makona-G3820, [coding-]complete genome^2^
	<SOURCE ORGANISM> *= Zaire ebolavirus*^1^
	</isolate> = Ebola virus/H.sapiens-wt/SLE/2014/Makona-G3820
	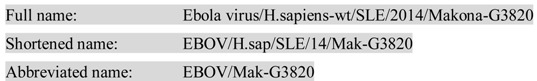
KM233095	<DEFINITION LINE> *= Zaire ebolavirus*^1^ isolate Ebola virus/H.sapiens-wt/SLE/2014/Makona-G3821, [coding-]complete genome^2^
	<SOURCE ORGANISM> *= Zaire ebolavirus*^1^
	</isolate> = Ebola virus/H.sapiens-wt/SLE/2014/Makona-G3821
	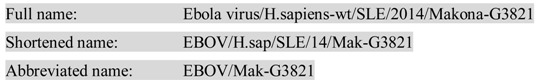
KM233096	<DEFINITION LINE> *= Zaire ebolavirus*^1^ isolate Ebola virus/H.sapiens-wt/SLE/2014/Makona-G3822, [coding-]complete genome^2^
	<SOURCE ORGANISM> *= Zaire ebolavirus*^1^
	</isolate> = Ebola virus/H.sapiens-wt/SLE/2014/Makona-G3822
	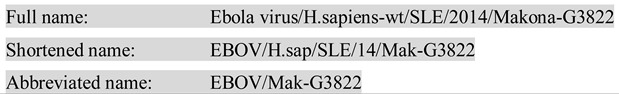
KM233097	<DEFINITION LINE> *= Zaire ebolavirus*^1^ isolate Ebola virus/H.sapiens-wt/SLE/2014/Makona-G3823, [coding-]complete genome^2^
	<SOURCE ORGANISM> *= Zaire ebolavirus*^1^
	</isolate> = Ebola virus/H.sapiens-wt/SLE/2014/Makona-G3823
	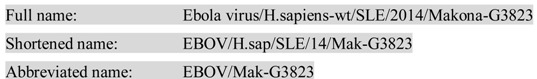
KM233098	<DEFINITION LINE> *= Zaire ebolavirus*^1^ isolate Ebola virus/H.sapiens-wt/SLE/2014/Makona-G3825.1, [coding-]complete genome^2^
	<SOURCE ORGANISM> *= Zaire ebolavirus*^1^
	</isolate> = Ebola virus/H.sapiens-wt/SLE/2014/Makona-G3825.1
	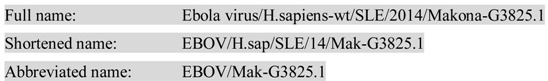
KM233099	<DEFINITION LINE> *= Zaire ebolavirus*^1^ isolate Ebola virus/H.sapiens-wt/SLE/2014/Makona-G3825.2, [coding-]complete genome^2^
	<SOURCE ORGANISM> *= Zaire ebolavirus*^1^
	</isolate> = Ebola virus/H.sapiens-wt/SLE/2014/Makona-G3825.2
	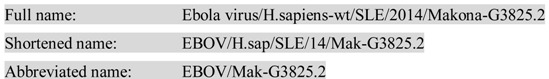
KM233100	<DEFINITION LINE> *= Zaire ebolavirus*^1^ isolate Ebola virus/H.sapiens-wt/SLE/2014/Makona-G3826, [coding-]complete genome^2^
	<SOURCE ORGANISM> *= Zaire ebolavirus*^1^
	</isolate> = Ebola virus/H.sapiens-wt/SLE/2014/Makona-G3826
	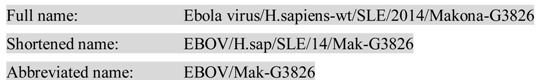
KM233101	<DEFINITION LINE> *= Zaire ebolavirus*^1^ isolate Ebola virus/H.sapiens-wt/SLE/2014/Makona-G3827, [coding-]complete genome^2^
	<SOURCE ORGANISM> *= Zaire ebolavirus*^1^
	</isolate> = Ebola virus/H.sapiens-wt/SLE/2014/Makona-G3827
	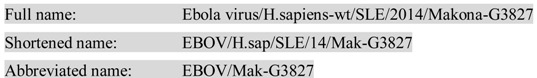
KM233102	<DEFINITION LINE> *= Zaire ebolavirus*^1^ isolate Ebola virus/H.sapiens-wt/SLE/2014/Makona-G3829, [coding-]complete genome^2^
	<SOURCE ORGANISM> *= Zaire ebolavirus*^1^
	</isolate> = Ebola virus/H.sapiens-wt/SLE/2014/Makona-G3829
	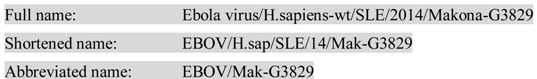
KM233103	<DEFINITION LINE> *= Zaire ebolavirus*^1^ isolate Ebola virus/H.sapiens-wt/SLE/2014/Makona-G3831, [coding-]complete genome^2^
	<SOURCE ORGANISM> *= Zaire ebolavirus*^1^
	</isolate> = Ebola virus/H.sapiens-wt/SLE/2014/Makona- G3831
	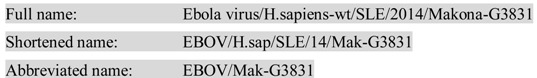
KM233104	<DEFINITION LINE> *= Zaire ebolavirus*^1^ isolate Ebola virus/H.sapiens-wt/SLE/2014/Makona-G3834, [coding-]complete genome^2^
	<SOURCE ORGANISM> *= Zaire ebolavirus*^1^
	</isolate> = Ebola virus/H.sapiens-wt/SLE/2014/Makona-G3834
	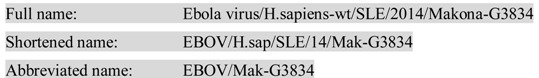
KM233105	<DEFINITION LINE> *= Zaire ebolavirus*^1^ isolate Ebola virus/H.sapiens-wt/SLE/2014/Makona-G3838, [coding-]complete genome^2^
	<SOURCE ORGANISM> *= Zaire ebolavirus*^1^
	</isolate> = Ebola virus/H.sapiens-wt/SLE/2014/Makona-G3838
	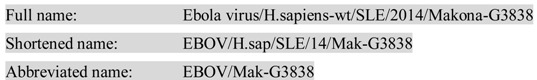
KM233106	<DEFINITION LINE> *= Zaire ebolavirus*^1^ isolate Ebola virus/H.sapiens-wt/SLE/2014/Makona-G3840, [coding-]complete genome^2^
	<SOURCE ORGANISM> *= Zaire ebolavirus*^1^
	</isolate> = Ebola virus/H.sapiens-wt/SLE/2014/Makona-G3840
	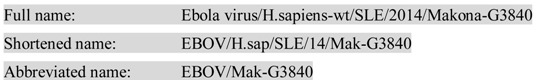
KM233107	<DEFINITION LINE> *= Zaire ebolavirus*^1^ isolate Ebola virus/H.sapiens-wt/SLE/2014/Makona-G3841, [coding-]complete genome^2^
	<SOURCE ORGANISM> *= Zaire ebolavirus*^1^
	</isolate> = Ebola virus/H.sapiens-wt/SLE/2014/Makona-G3841
	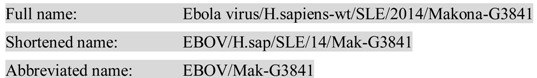
KM233108	<DEFINITION LINE> *= Zaire ebolavirus*^1^ isolate Ebola virus/H.sapiens-wt/SLE/2014/Makona-G3845, [coding-]complete genome^2^
	<SOURCE ORGANISM> *= Zaire ebolavirus*^1^
	</isolate> = Ebola virus/H.sapiens-wt/SLE/2014/Makona-G3845
	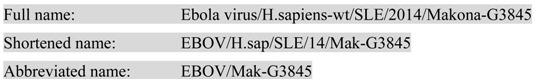
KM233109	<DEFINITION LINE> *= Zaire ebolavirus*^1^ isolate Ebola virus/H.sapiens-wt/SLE/2014/Makona-G3846, [coding-]complete genome^2^
	<SOURCE ORGANISM> *= Zaire ebolavirus*^1^
	</isolate> = Ebola virus/H.sapiens-wt/SLE/2014/Makona-G3846
	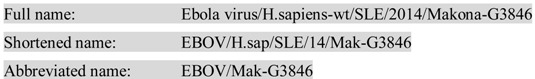
KM233110	<DEFINITION LINE> *= Zaire ebolavirus*^1^ isolate Ebola virus/H.sapiens-wt/SLE/2014/Makona-G3848, [coding-]complete genome^2^
	<SOURCE ORGANISM> *= Zaire ebolavirus*^1^
	</isolate> = Ebola virus/H.sapiens-wt/SLE/2014/Makona-G3848
	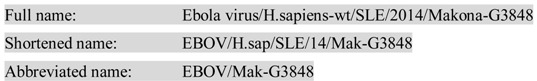
KM233111	<DEFINITION LINE> *= Zaire ebolavirus*^1^ isolate Ebola virus/H.sapiens-wt/SLE/2014/Makona-G3850, [coding-]complete genome^2^
	<SOURCE ORGANISM> *= Zaire ebolavirus*^1^
	</isolate> = Ebola virus/H.sapiens-wt/SLE/2014/Makona-G3850
	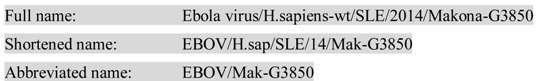
KM233112	<DEFINITION LINE> *= Zaire ebolavirus*^1^ isolate Ebola virus/H.sapiens-wt/SLE/2014/Makona-G3851, [coding-]complete genome^2^
	<SOURCE ORGANISM> *= Zaire ebolavirus*^1^
	</isolate> = Ebola virus/H.sapiens-wt/SLE/2014/Makona-G3851
	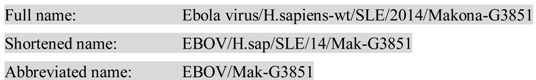
KM233113	<DEFINITION LINE> *= Zaire ebolavirus*^1^ isolate Ebola virus/H.sapiens-wt/SLE/2014/Makona-G3856.1, [coding-]complete genome^2^
	<SOURCE ORGANISM> *= Zaire ebolavirus*^1^
	</isolate> = Ebola virus/H.sapiens-wt/SLE/2014/Makona-G3856.1
	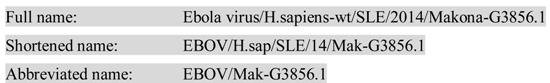
KM233114	<DEFINITION LINE> *= Zaire ebolavirus*^1^ isolate Ebola virus/H.sapiens-wt/SLE/2014/Makona-G3856.3, [coding-]complete genome^2^
	<SOURCE ORGANISM> *= Zaire ebolavirus*^1^
	</isolate> = Ebola virus/H.sapiens-wt/SLE/2014/Makona-G3856.3
	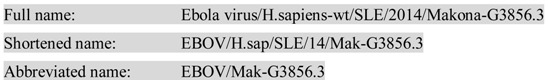
KM233115	<DEFINITION LINE> *= Zaire ebolavirus*^1^ isolate Ebola virus/H.sapiens-wt/SLE/2014/Makona-G3857, [coding-]complete genome^2^
	<SOURCE ORGANISM> *= Zaire ebolavirus*^1^
	</isolate> = Ebola virus/H.sapiens-wt/SLE/2014/Makona-G3857
	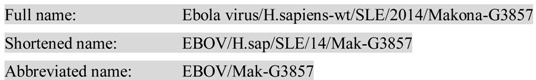
KM233116	<DEFINITION LINE> *= Zaire ebolavirus*^1^ isolate Ebola virus/H.sapiens-wt/SLE/2014/Makona-NM042.1, [coding-]complete genome^2^
	<SOURCE ORGANISM> *= Zaire ebolavirus*^1^
	</isolate> = Ebola virus/H.sapiens-wt/SLE/2014/Makona-NM042.1
	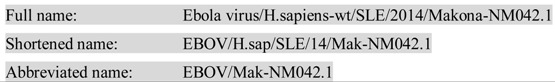
KM233117	<DEFINITION LINE> *= Zaire ebolavirus*^1^ isolate Ebola virus/H.sapiens-wt/SLE/2014/Mak-NM042.2, [coding-]complete genome^2^
	<SOURCE ORGANISM> *= Zaire ebolavirus*^1^
	</isolate> = Ebola virus/H.sapiens-wt/SLE/2014/Makona-NM042.2
	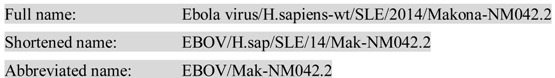
KM233118	<DEFINITION LINE> *= Zaire ebolavirus*^1^ isolate Ebola virus/H.sapiens-wt/SLE/2014/Makona-NM042.3, [coding-]complete genome^2^
	<SOURCE ORGANISM> *= Zaire ebolavirus*^1^
	</isolate> = Ebola virus/H.sapiens-wt/SLE/2014/Makona-NM042.3
	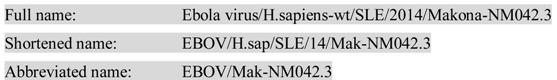
KM251803	<DEFINITION LINE> *= Zaire ebolavirus*^1^ isolate Ebola virus/H.sapiens-frag/NGA/2014/Makona-01072014 L gene, partial CDS^3^
	<SOURCE ORGANISM> *= Zaire ebolavirus*^1^
	<strain> = [EMPTY]
	</isolate> = Ebola virus/H.sapiens-frag/NGA/2014/Makona-01072014
	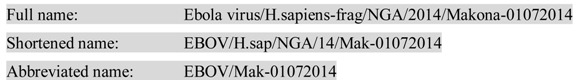

^1^ Neither RefSeq nor GenBank currently can handle italics, which is why the species names are not italicized in the actual entry’s definition line and <organism> fields. ^2^ The International Nucleotide Sequence Database Collaboration (INSDC) standard currently does not offer options other than “complete” or “partial,” and, in particular, does not provide a possibility for the designation “coding-complete.” We, therefore recommend using “complete” for what is actually “coding-complete” as the word “partial” implies short sequence fragments to most users. ^3^ CDS = coding sequence.

## 4. The 2014 Ebola Virus Variant Originating in the Democratic Republic of the Congo

**Figure 2:**
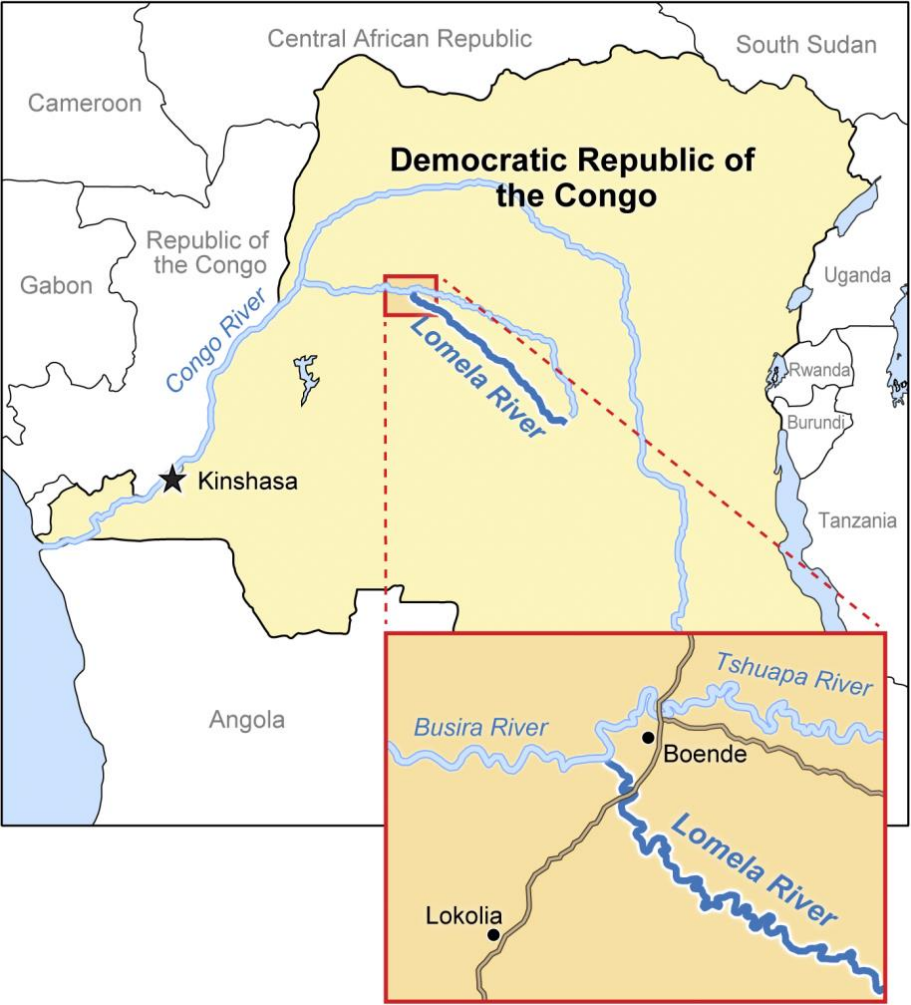
Location of the Lomela River.

In late 2014, EVD broke out in Boende District, Democratic Republic of the Congo (3-letter country code: COD), Middle Africa. Epidemiological and phylogenetic studies indicate that this limited EVD outbreak was caused by a single introduction of one particular ebolavirus, Ebola virus (EBOV), into humans (*Homo sapiens*) from an unknown reservoir and therefore that all subsequent several cases (almost 70) are derived from one unnamed variant [9]. Preliminary clinical observations among EVD patients in this outbreak do not contradict past descriptions of EVD [9], *i.e.*, this novel unnamed EBOV variant is not a strain as defined in [10]. Here we propose the name “Lomela” (IPA: [lɔ'mɛlɑ] or [lɔ'mɛlə]; English phonetic notation: law-**me**-lah or law-**me**-l*uh*) after the Lomela River that runs through COD’s Boende District (see Figure 2) as the variant name for this Middle African virus. The general name for the 2014 Middle African virus is therefore


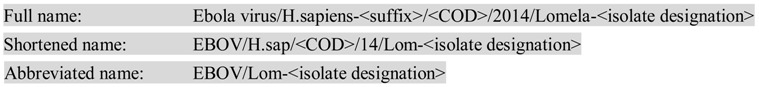


Accordingly, in all currently deposited sequences of EBOV/Lom, the definition line will be adjusted to “*Zaire ebolavirus* isolate Ebola virus/H.sapiens-<suffix>/<COD>/2014/Lomela-<isolate designation>, with the <suffix> and <isolate designation> fields will be filled according to their origin. The GenBank <strain> field will be cleared throughout; the Genbank <isolate> field will be filled with “Ebola virus/H.sapiens-<suffix>/<COD>/2014/Lomela-<isolate designation>”, and the <organism> field will be corrected, if necessary, to “*Zaire ebolavirus*” (Table 4).

**Table 4:** EBOV isolates from the Democratic Republic of the Congo Boende EVD outbreak, 2014: Updated and/or corrected GenBank information and final names

GenBank Accession Number	<GenBank field> = Updated/corrected information

	Final names
KM517570	<DEFINITION LINE> *= Zaire ebolavirus*^1^ isolate Ebola virus/H.sapiens-wt/GIN/2014/Makona-C15, [coding-]complete genome^2^
	<SOURCE ORGANISM> *= Zaire ebolavirus*^1^
	</isolate> = Ebola virus/H.sapiens-frag/COD/2014/Lomela-Lokolia16
	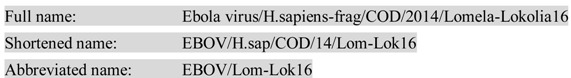
KM517571	<DEFINITION LINE> *= Zaire ebolavirus*^1^ Ebola isolate virus H.sapiens-frag/COD/2014/Lomela-Lokolia17 L gene, partial CDS^2^
	<SOURCE ORGANISM> *= Zaire ebolavirus*^1^
	</isolate> = Ebola virus/H.sapiens-frag/COD/2014/Lomela-Lokolia17
	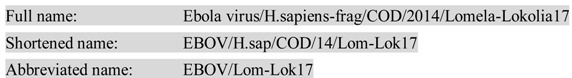
KM519951	<DEFINITION LINE> *= Zaire ebolavirus*^1^ Ebola isolate virus H.sapiens-wt/COD/2014/Lomela-Lokolia1, [coding-]complete genome^3^
	<SOURCE ORGANISM> *= Zaire ebolavirus*^1^
	</isolate> = Ebola virus/H.sapiens-wt/COD/2014/Lomela-Lokolia1
	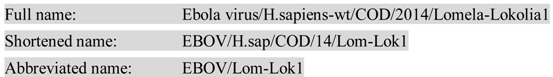

^1^ Neither RefSeq nor GenBank currently can handle italics, which is why the species names are not italicized in the actual entry’s definition line and <organism> fields. ^2^ CDS = coding sequence ^3^ The International Nucleotide Sequence Database Collaboration (INSDC) standard currently does not offer options other than “complete” or “partial,” and, in particular, does not provide a possibility for the designation “coding-complete.” We, therefore recommend using “complete” for what is actually “coding-complete” as the word “partial” implies short sequence fragments to most users. Note: “Lokolia” – IPA: [lɔ'kɔliə] or [lə'kɔliə]; English phonetic notation: law-**kaw**-lee-*uh* or luh-**kaw**-lee-*uh*.
